# Small Extracellular Vesicles in the Development, Diagnosis, and Possible Therapeutic Application of Esophageal Squamous Cell Carcinoma

**DOI:** 10.3389/fonc.2021.732702

**Published:** 2021-08-30

**Authors:** Zheng Zhao, Shuyue Yang, Anni Zhou, Xiao Li, Rui Fang, Shutian Zhang, Guiping Zhao, Peng Li

**Affiliations:** Department of Gastroenterology, Beijing Friendship Hospital, Capital Medical University, Beijing, China

**Keywords:** esophageal squamous cell carcinoma, exosome, exosomal RNAs, biomarker, diagnosis

## Abstract

Esophageal squamous cell carcinoma (ESCC) persists among the most lethal and broad-spreading malignancies in China. The exosome is a kind of extracellular vesicle (EV) from about 30 to 200 nm in diameter, contributing to the transfer of specific functional molecules, such as metabolites, proteins, lipids, and nucleic acids. The paramount role of exosomes in the formation and development of ESCC, which relies on promoting intercellular communication in the tumor microenvironment (TME), is manifested with immense amounts. Tumor-derived exosomes (TDEs) participate in most hallmarks of ESCC, including tumorigenesis, invasion, angiogenesis, immunologic escape, metastasis, radioresistance, and chemoresistance. Published reports have delineated that exosome-encapsulated cargos like miRNAs may have utility in the diagnosis, as prognostic biomarkers, and in the treatment of ESCC. This review summarizes the function of exosomes in the neoplasia, progression, and metastasis of ESCC, which improves our understanding of the etiology and pathogenesis of ESCC, and presents a promising target for early diagnostics in ESCC. However, recent studies of exosomes in the treatment of ESCC are sparse. Thus, we introduce the advances in exosome-based methods and indicate the possible applications for ESCC therapy in the future.

## Introduction

Esophageal cancer ranks the seventh most prevalent malignancy and the sixth-highest cancer-related mortality globally (1). Esophageal cancer is broadly divided into esophageal squamous cell carcinoma (ESCC) and esophageal adenocarcinoma (2). The incidence of esophageal cancer in men is approximately three to four times that in women and varies among countries (3). The highest rates of ESCC are found in Eastern Asia, where ESCC accounts for more than 90% of esophageal cancers (4). Despite significant progress in diagnosis and treatment, ESCC is often identified late, which leads to delayed treatment. The reason for this challenge can be attributable to multiple aspects: in the early stage, ESCC is characterized by a lack of specific symptoms and definitive diagnosis; coming to the advanced stage, ESCC can exhibit considerable metastatic potential and strong resistance to conventional treatment. Given the above, ESCC is commonly presented with a poor prognosis, as the 5-year survival rate of late-stage ESCC is approximately 10–20% (5). Hence, more effort is urgently needed to reveal the mechanisms of tumorigenesis and increase the early diagnosis rate of ESCC.

In the last decade, there has been a steep increase in the investigations focusing on extracellular vesicles’ (EVs’) physiological and pathological functions, referring to multiple subtypes of cell-released, membranous structures (6–8). In particular, exosome is among the most studied and deliberated population of EVs in the rapidly growing number of publications. Notably, MISEV2018 guidelines have endorsed that the term “exosome” should be applied strictly to an EV of endosomal origin owing to the difficulties to confirm such an origin after an EV has left the cell (8, 9). According to the conventional description of exosome (from about 30 to 200 nm in diameter) (10), it would be more appropriate to nominate “exosome” as “small extracellular vesicle (sEV)”, as the guidelines propose. However, considering the number of studies published before the criteria were issued, we decide to preserve “exosome”, referring to “small extracellular vesicle of endosomal origin”, in this review to help readers adapt to the new standard.

With molecular heterogeneity, exosomes encapsulate diverse bioactive molecules, ranging from nucleic acid (including DNA and RNA) to proteins, lipids, and other metabolites (11). Exosomes have mediated a new paradigm of intercellular communication *via* the transfer of bioactive molecules from donor cells to recipient cells, and they function in both normal physiology and acquired pathological activities, such as reproduction, immune responses, metabolic and cardiovascular diseases, ischemic diseases, neurodegeneration, and malignant tumors (12–17). In the process of tumorigenesis, exosomes can participate in the formation of the tumor microenvironment (TME), the proliferation of cancer cells, angiogenesis, metastasis, therapy resistance, and many other physiological and pathological processes (18). The amounts and cargos of exosomes derived from the same cell can dramatically vary from different conditions, and the heterogeneity of exosomal cargos has been recognized among different individuals (19–21). Considering the homogeneity between exosomes and donor cells, these cargos of the tumor-derived exosomes (TDEs) carry cancer-related information and allow them to fulfill diagnostic functions, serving as tumor biomarkers of ESCC that can be detected in early-stage cancer (22, 23). Moreover, the specialty of exosomes in delivering diverse and specific functional cargos into recipient cells has accelerated their clinical application in the therapy of patients with malignant tumors or other diseases (24–26). Ongoing studies and trials have proven that exosomes can be engineered to carry specific lipids, proteins, and other chemotherapeutic agents to targeted cells or organs and facilitate the treatment of several diseases (27–30).

**Figure 1:**
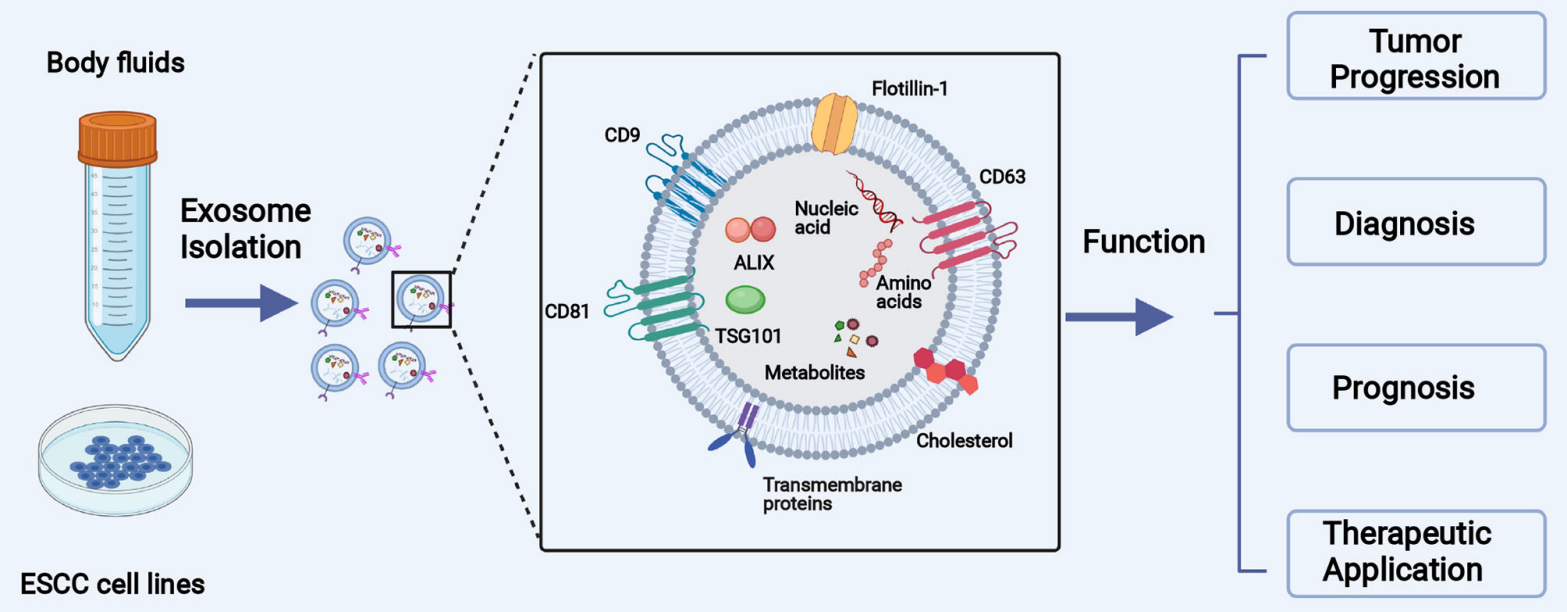
The main objective of this review is to introduce the biology of exosomes, summarize the function of exosomal cargos in the initiation and development of ESCC, and collect the clinical application of exosomes in the diagnosis and treatment of ESCC.

The primary objectives of this review are to introduce the biology of exosomes, summarize the function of exosome-carried cargos in the initiation and development of ESCC, and discuss the potential clinical applications in both the early diagnosis and treatment of ESCC (**Figure 1**).

## Biology of Exosome (sEVs)

Exosomes, which should have been called sEVs following MISEV2018 guidelines, are a kind of lipid bilayer-encapsulated, nanosized vesicles that are enriched in specific DNA, RNA, lipids, proteins, and bioactive compounds (10).

**Figure 2:**
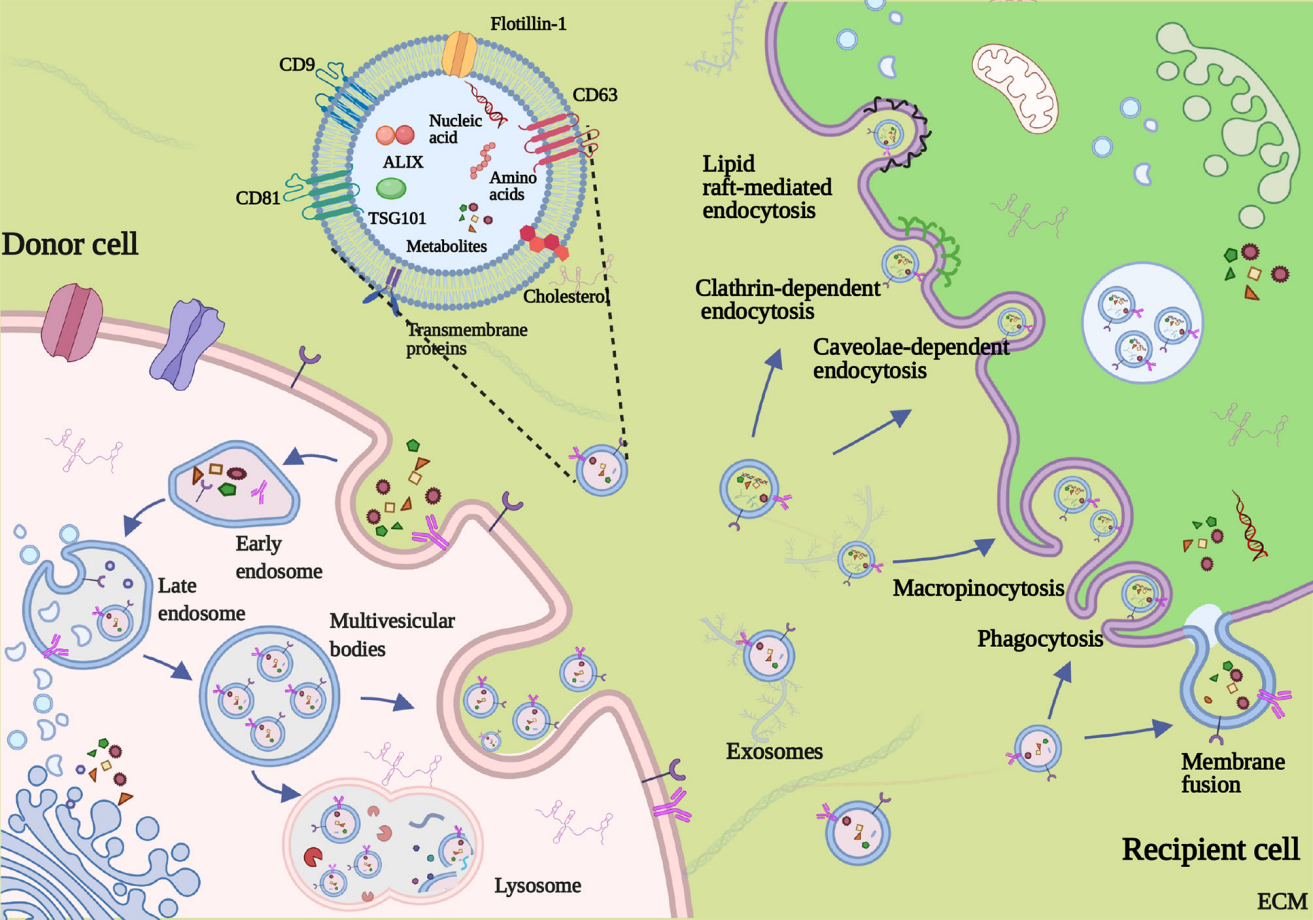
Formation, secretion, and uptake of exosome. Invagination of the plasma membrane forms the early endosome. Then, inward invagination of endosomes gives birth to the formation of multivesicular bodies (MVBs) containing intraluminal vesicles (ILVs). Exosomes are eventually released by fusing of MVBs to the plasma membrane and the exocytosis of ILVs. The mechanism of exosome uptake includes direct fusing with the plasma membrane, macropinocytosis, phagocytosis, caveolin-mediated, lipid raft-mediated endocytosis, and clathrin-dependent endocytosis.

Generally, the biogenesis and releasing of exosomes involve the double invagination of the plasma membrane and the sequential generation of multivesicular bodies (MVBs) and intraluminal vesicles (ILVs) (11) (**Figure 2**). The first invagination of the plasma membrane forms a cup-shaped structure, containing cell-surface proteins and soluble proteins from the extracellular milieu. Then, the cup-shaped plasma membrane buds in the inner side of the cell, which gives rise to an early-sorting endosome (ESE) and, sometimes, may directly fuse with a preexisting ESE. Meanwhile, the endoplasmic reticulum (ER), mitochondria, and trans-Golgi network (TGN) also engage with the formation of the ESEs. Furthermore, the ESEs can also blend into the ER and TGN, possibly interpreting how the extracellular and cell-surface ingredients enter them (31–36). Afterward, ESEs form late-sorting endosomes (LSEs) and subsequently give rise to MVBs (also named as multivesicular endosomes). MVBs come into being with the inward invagination of the endosomal limiting membrane, the double invagination of the plasma membrane exactly, and they will be released as intraluminal vesicles (ILVs) after fusion with the plasma membrane. During the process, cytoplasmic constituents can enter the newly forming ILVs, leading to further changes in the future exosomal cargos (32, 37). Some proteins, including endosomal sorting complexes required for transport proteins (ESCRT), soluble N-ethylmaleimide-sensitive factor attachment protein receptors (SNAREs), apoptosis-linked gene 2-interacting protein X (ALIX), tumor susceptibility gene 101 (TSG101), Rab GTPases, CD9, CD63, and CD8 1, play a critical part in the origin and biogenesis of exosomes, and some are regarded as markers of exosomes (38, 39). After being secreted into the extracellular milieu, exosomes are delivered and identified by the targeted recipient cells. As a result, they alter the phenotype and biological response of these recipient cells (40). The mechanism of exosome uptake is complex; the fate of the exosomal contents and the molecular alterations induced in recipient cells add complexity to the cell–cell crosstalk (41). When docking the recipient cell, exosomes can activate signaling pathways by straightly interacting with the receptors on the cell surface, directly fusing with the plasma membrane, or getting internalized (42). Firstly, the interaction between exosomes and extracellular receptors has been reported to exist in mediating immunomodulatory. For example, Tkach et al. showed that exosomes secreted by dendritic cells (DCs) could carry MHC–peptide complexes and bind Toll-like receptor ligands on the bacterial surface, which induced the activation of bystander DCs and T lymphocytes (43, 44). Besides, the families of SNAREs and Rab proteins were reported to mediate the fusion with the plasma membrane and release exosomal cargos (45). Moreover, as representative of internalization, the mode of clathrin-mediated endocytosis has been demonstrated in multiple cell types, such as gastric epithelial cells, colon tumor cells, and cardiomyocytes (46–48). Different exosomal uptake modes may be attributed to the properties of the exosome that shuttles cargos and the metabolic status of recipient cells, but the precise regulating mechanism deserves additional in-depth exploration.

## Roles of Exosome in the Initiation and Development of ESCC

**Figure 3:**
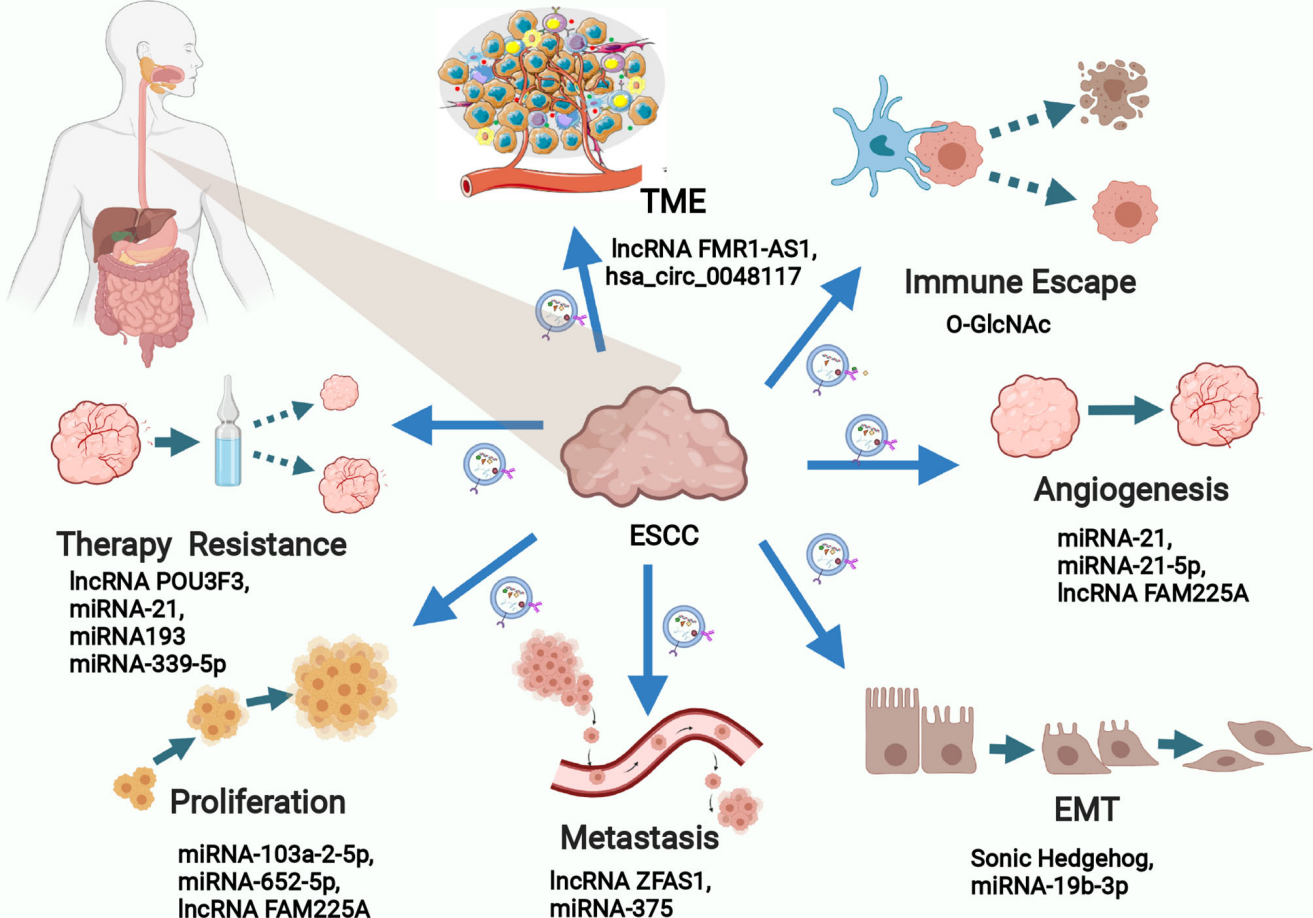
The biological function of exosomal cargos in the development of ESCC.

Exosome-related research has focused on the exosome’s ability to efficiently transfer an array of selected cargos to recipient cells (49). Studies about the function of exosomes in malignant tumors have developed substantially compared with studies in other fields, and increasing evidence supports exosome-mediated intercellular crosstalk in the TME (50, 51). Accumulating evidence has revealed that exosomes are involved in many features of malignant tumors, including neoplasia, progression, metastasis, angiogenesis, and drug resistance (52–55). In recent years, research involving the correlation between exosomes and ESCC has increased rapidly and yielded valuable information about the function of exosomes in ESCC progression. Here, we summarize the biological function of exosomes that shuttle cargos in the initiation and development of ESCC (**Figure 3**), as shown in **Table 1**. Understanding the function of exosomes and how to use exosomes in ESCC cells to transfer nanoparticles in cell–cell communication are topics at the forefront of oncobiology and may open new avenues for ESCC treatment.

**Table 1:** Roles of exosome in the initiation and development of ESCC.

Type	Molecule	Function	Signaling/Target	Ref.
miRNA	miRNA-19b-3p	Reduce apoptosis rate, and promote migration and invasion	PTEN	(56)
	miRNA-103a-2-5p	Promote proliferation and migration	CDH11 gene and NR3C1 gene	(57)
	miRNA-652-5p	Inhibit proliferation and metastasis	PARG and VEGF pathways	(58)
	miRNA-21-5p	Promote angiogenesis	PDCD4 and PTEN/Akt pathway	(59)
	miRNA-21	Promote angiogenesis	SPRY1	(60)
		Promote chemotherapy resistance	PDCD4	(61)
	miRNA-375	Promote apoptosis, and inhibit proliferation, invasion, migration	ENAH	(62)
	miRNA-193	Promote chemotherapy resistance	VEGF and Jak-STAT pathways	(63)
	miRNA-339-5p	Enhance radiosensitivity	Cdc25A	(64)
lncRNA	FMR1-AS1	Maintain TME, promote proliferation, invasion, and inhibit apoptosis	TLR7/NFκB/c-Myc pathway	(65)
	ZFAS1	Promote proliferation, migration, invasion, and inhibit apoptosis	miRNA-124/STAT3 axis	(66)
	FAM225A	Promote apoptosis, and inhibit proliferation, migration, and invasion	miRNA-206/NETO2/FOXP1	(67)
	UCA1	Inhibit proliferation, invasion and migration	miRNA-613	(68)
	PART1	Promote chemotherapy resistance	miRNA-129/Bcl-2 pathway	(69)
	POU3F3	Promote proliferation and chemotherapy resistance	IL-6	(70)
circRNA	has_circ_0048117	Promote invasion and migration	miRNA-140/M2 macrophage	(71)
Others	Sonic Hedgehog	Promote proliferation and migration	Hedgehog pathway	(72)
	O-GlcNAc transferase	Promote the immune escape	PD-1	(73)
	HMGB1	Promote the immune escape	PD-1 positive TAMs	(74)

### Tumor Microenvironment (TME)

The occurrence of ESCC is a result of a continuous accumulation of mutations in esophageal cells and oncogenic alteration in the TME (75). The TME involves blood vessels, the extracellular matrix (ECM), cytokines, and stromal cells and is indispensable in tumorigenesis because it provides necessary conditions for tumor growth and manipulates the interaction between cancer cells and their surroundings (76). Exosomes act as an essential role in the formation and reprogramming of the TME, as has been widely documented in many cancer types (77–79). In ESCC, Li et al. found that exosomal FMR1 antisense RNA 1 (FMR1-AS1) could remodel the TME in ESCC (65). The study confirmed that the FMR1-AS1 exosomes were secreted from cancer stem-like cells (CSCs) of ESCC, which transferred stemness phenotypes to recipient non-CSCs in the TME through the mechanism of activating toll-like receptor 7-nuclear factor κ B (TLR7-NFκB) signaling, upregulating the c-Myc level in recipient cells. Another example of exosomal cargos promoting the TME formation involves circ-0048117-rich exosomes derived from hypoxic ESCC cells, promoting M2 macrophage polarization to alter the components in the microenvironment (71). Researchers indicated that hypoxic exosomes modulated the TME in ESCC *via* the transformation of endothelial cell phenotypes and transcriptomes, which enhance angiogenesis and metastasis. Moreover, the exchange of exosomes in cancer cells and the stroma is bidirectional, and cancer-associated fibroblasts (CAFs) can also secrete exosomal Sonic Hedgehog to promote the generation of TME in ESCC (72). These findings suggest that exosomes can play an essential role in the formation, remolding, and normal function of the TME and that novel therapies targeting the TME may be a new approach to cancer treatment.

### Proliferation and Apoptosis

The progression of ESCC results from rapid growth and expansion of cancer cells, which may incur tumor survival and defiance to therapy. Exosomes can influence the growth of ESCC by mediating the apoptosis, cycle, and proliferation rate of ESCC cells (80). Molecular profiling has indicated that exosomal miRNA-19b-3p from EC9706 cells targets *PTEN*, a well-known tumor suppressor gene, to regulate the apoptosis of ESCC (56). A similar study elucidated that exosomal lncRNA ZNFX1 antisense RNA 1 (ZFAS1) derived from EC109 cells regulates ESCC proliferation, apoptosis, and migration *via* targeting the miRNA-124/STAT3 signaling pathway (66). Cancer cell-derived exosomes can regulate the ratio of G1-phase cells and influence the cycle and migration ability of ESCC cells (81). Some researchers have demonstrated that the proliferation and apoptosis of ESCC cells are modulated by several other exosomal cargos, including miRNA-103a-2-5p, miRNA-652-5p, lncRNA Family with sequence similarity 225 member A (FAM225A), and lncRNA urothelial cancer-associated 1 (UCA1) (57, 58, 67, 68). Generally, these exosomal contents can work by mediating the expression of proliferation- or apoptosis-related proteins and triggering a subsequent signaling pathway. Importantly, these studies also indicated that the identified exosomal RNAs and other exosomal contents can facilitate the proliferation ability of tumor cells alone and may concurrently alter the potential for migration, angiogenesis, and metastasis. Above all, the activation of cell proliferation cannot entirely be attributed to the expansion and development of ESCC; instead, it results from several steps, including migration and metastasis, angiogenesis, immune response, and therapy resistance. The role of exosomes in these steps of tumor development is explored in the following sections.

### Angiogenesis

Angiogenesis, a critical phase during neoplasia, migration, and metastasis, is a multistep formation of neovascularization through which cancer cells obtain sufficient oxygen, nutrition, and energy (82). Reports have illustrated that some exosomes could play a role in inducing angiogenesis in many cancer types (83). Exosomes can deliver numerous pro-angiogenic bioactive substances, including vascular endothelial growth factor (VEGF), miRNAs, or other bioactive mediators. Published data suggest that exosomal cargos accelerate angiogenesis by suppressing the expression of anti-angiogenesis genes and promoting the expression of pro-angiogenic genes (84, 85). For example, compared with normal exosomes, hypoxic exosomes have played a unique role in facilitating the aggressive behavior of human umbilical vein endothelial cells (HUVECs) both *in vitro* and *in vivo*, and HUVECs exposed to hypoxic exosomes induce enhanced proliferation, metastatic dissemination, and vessel formation ability in ESCC (86). Consistent with that study, Zhuang showed that ESCC cell-derived exosomal miRNA-21 potentiates the angiogenesis ability of HUVEC by targeting sprouty RTK signaling antagonist 1 (SPRY1) in ESCC (60). Moreover, exosomal miRNA-21-5p has been shown to significantly promote the angiogenesis of targeted cells *via* the activation of programmed cell death 4 and downgrading of the signaling pathway or the PTEN/Akt signal pathway in ESCC (59). Similar findings have suggested that exosomal lncRNA FAM225A accelerates ESCC angiogenesis by binding to miRNA-206 and promoting NETO2 and FOXP1 expression (67). Given the pivotal role of angiogenesis in ESCC development and progression, exosome-related research may provide a new avenue to counteract these mechanisms of progression in ESCC, and these discoveries will become even more promising if they are linked to antitumor vascular drugs.

### Epithelial-Mesenchymal Transition and Metastasis

Metastasis is a critical step in tumor growth, and it remains a paramount threshold for cancer treatment and the chief cause of cancer mortality (87). Metastasis is a complicated and intricate process involving several steps such as epithelial-mesenchymal transition (EMT) of cancer cells, migration and infiltration into surrounding tissues, intravascular transport, and recognition and establishment in distant tissues (88, 89). Intercellular communication by delivering exosomes from primary tumor cells to the local microenvironment or distant organs is crucial for the phenotypic change and biological aggressive behavior of cancer cells, forming a pre-metastatic niche, and attachment and implantation to distant organs (90). Esophageal cancer cell-derived exosomes can modulate gene expression of recipient cancer cells, leading to an increased risk of invasion and metastasis. For example, the Sonic Hedgehog (SHH) signaling pathway, which has played important roles during development and in cancer (91, 92), can drive tumorigenesis and progression of ESCC. One study showed that exosomal Sonic Hedgehog derived from cancer-associated fibroblasts (CAFs) could increase the activation of N-cadherin and Vimentin in EC109 cell lines and consequently promoted the growth and migration abilities of ESCC (72). Similarly, exosomes derived by infiltrating T cells from irradiated esophageal carcinoma can incur the EMT in ESCC and facilitate metastasis (93). Conversely, human umbilical cord mesenchymal stem cells can suppress enabled homolog (ENAH) expression and decrease the invasion and migration ability of ESCC *via* the exosomal delivery of miRNA-375 (62). Exosomes play a pivotal role in forming a premetastatic niche in distant organs in ESCC, like in gastric cancer and breast cancer (94). This role may be attributed partly to the predisposition of early lymphatic metastasis in ESCC; metastatic dissemination in the liver or lung is relatively rare (95). In conclusion, exosomal cargos can exert pro-tumorigenic effects in most steps of ESCC metastasis, thus promoting the metastatic potential of ESCC. The studies discussed here may offer new insights that help the researcher understand the function of exosomes in metastatic ESCC and uncover exosome-based therapies that may curb cancer metastasis.

### Immune Response and Therapy Resistance

The elimination of tumor cells relies heavily on the immune system *in vivo* and exogenous therapies, such as drugs or irradiation (96). The immune system is an intricate network that can guard the body by monitoring, recognizing, and eliminating foreign invaders, such as bacteria, parasites, and endogenous antigens like cancer cells (97). These days, when we talk about the relationship between immune response and tumors, programmed cell death protein 1 (PD-1) is one of the monumental works that are closely associated with it indeed (98). A recent study suggested that O-linked β-N-acetylglucosamine (O-GlcNAc) transferase from stem cells of ESCC can upregulate PD-1 in CD8+ T cells and promote cancer immunosuppression (73). Furthermore, exosomes isolated from serum, plasma, urine, or other body fluids of patients with ESCC, as well as from ESCC cell lines, can reduce B-cell proliferation and induce an increase in interleukin-10 positive regulatory B cells and a high level of PD-1 regulatory B cells (99). Besides, another research demonstrated that exosomal High Mobility Group Box 1 (HMGB1) obtained from ESCC could successfully trigger clonal expansion of PD1 positive tumor-associated macrophages (TAMs), which thereby created conditions for the development of ESCC (74). These findings above contribute to understanding exosomal functions in the immune response and illustrate exosomes’ therapeutic application that promotes antitumor immune responses.

Currently, chemotherapy is regarded as the most effective therapy for ESCC after surgery, and tumor recurrence can be attributed mainly to chemotherapy resistance (100, 101). Tumors can achieve drug resistance in many ways, including *via* information exchange by exosomes (102). A recent study indicated that exosomes carrying lncRNA prostate androgen-regulated transcript 1 (PART1) derived from Gefitinib-resistant cells confer cisplatin resistance in ESCC (69). Furthermore, several studies have elucidated that many other exosome-shuttled cargos, such as lncRNA POU class 3 homeobox 3 (POU3F3), miRNA-21, and miRNA-193, are involved in Cisplatin resistance in ESCC (61, 63, 70). Apart from roles in chemoresistance, exosomes reportedly regulate radiation therapy and induce radiation-induced bystander effect (RIBE) (103). Exosomal miRNA-339-5p can mediate the radiosensitivity of ESCC by downregulating cell division cycle 25A (Cdc25A) and can predict outcomes in preoperative radiotherapy (64). These discoveries capture the role of exosomes in therapy resistance and shed light on how engineered exosomes may deliver therapeutic agents for ESCC treatment in the future.

## Clinical Application of Exosome in ESCC

### Diagnostic Potential of Exosomal Cargos as Biomarkers for ESCC

ESCC is considered silent cancer because it lacks characteristic manifestations in the early stage. Patients are frequently diagnosed in middle or late stages, delaying the optimal time for treatment and causing a high mortality rate (104). Therefore, it is paramount to find early diagnostic methods to identify patients with ESCC to benefit from early interventions (105). Currently, the gold standard for the diagnosis of ESCC is tissue biopsy under endoscopy, an invasive inspection with correspondingly high costs (106). A non-invasive diagnostic method in early-stage ESCC is urgently needed. Researchers have made great efforts to screen for ESCC biomarkers; possible candidates include circulating tumor cells (CTCs), serum miRNAs, small extracellular vesicles (sEVs), as well as circulating tumor DNA (ctDNA) (107, 108). Exosomal cargos serve as promising tumor biomarkers because they reflect the donor cell and their presence in various biological fluids (109–111). New research into the potential application of exosomes as tumor biomarkers has emerged and yielded valuable information for additional in-depth exploration (112, 113). Growing evidence has confirmed that exosomal RNAs outperform peripheral blood-free RNAs in cancer diagnosis because of several advantages: First, exosomes exist in all biological fluids and are easily accessible compared with plasma. Second, exosomal cargos can be well protected from degradation by enzymes or elimination by the biological barrier. Third, the components of exosomes have high homology with donor cells, which may encourage a higher specificity of exosome-based detection. Last, the concentration of exosomal cargos is higher than the expression of plasma RNAs (114–116). Here, we focus on state-of-the-art exosomal cargos in ESCC.

The distinct expression of exosome-shuttling contents between cancer cells and normal cells supports the application of exosomes as biomarkers for ESCC. Among these exosomal compounds, exosome-carrying miRNAs are most investigated. For example, Zeng corroborated that exosome-shuttled miRNA-19b-3p separated from patients with ESCC is significantly upregulated compared with healthy controls, suggesting that serum exosome-encapsulated miRNA-19b-3p highlights the potential utility of exosomal RNAs in the early detection of ESCC (56). A study from 51 patients with ESCC and 41 with benign diseases showed that plasma exosomal miRNA-21 levels were significantly elevated in ESCC versus benign diseases so that they were suitable to be biomarkers for early diagnosis of ESCC (117). Except for the effect of exosomes on the differential diagnosis of ESCC, lymph node metastasis and TNM grade have been associated with the expression of some exosomal cargos that may serve as independent prognostic factors of ESCC. Lu et al. suggested that tumor cells-derived exosomes markedly upregulated the expression of hsa-circ-0048117 under the condition of hypoxia—a change that may be positively correlated with advanced T and N stages serves as a biomarker for progression (71). Similar studies have revealed that downregulated exosomal miRNA-339-5p and miRNA-652-5p in the serum are related to advanced TNM stages and a higher lymph node metastasis rate (58, 64). Moreover, several studies have indicated that higher serum exosomal miRNA-182, miRNA-766-3p, lncRNA POU3F3, and has-circ-0026611 levels in patients with ESCC are positively related to poor prognosis (70, 118–120). Except for exosomal RNAs, a small amount of exosome-carrying protein has been reported previously. The over-expression of Stathmin-1, regarded as microtubule depolymerization protein, is related to the process of tumor spread, adverse clinical outcomes, and chemoresistance in many types of cancer, especially squamous cell carcinoma, by controlling cell division, proliferation, and migration (121–124). In ESCC, Yan et al. corroborated that the average expression of stathmin-1 elevated in oncogenic exosomes, and the serum stathmin-1 level in patients with ESCC was obviously higher than that of healthy individuals (125). In addition, elevated concentration of stathmin-1 was related to lymphatic metastasis and late staged cancer.

Thus, several exosomal cargos have been identified as biomarkers for ESCC in applying possible diagnosis and potential prognosis, as described in **Table 2**. It is noteworthy that few literature reports address the diagnostic value of these molecules; the diagnostic efficacy of most candidates, apart from few miRNAs like miRNA-21, deserve additional validation. Much work remains before exosomal cargos can be applied as ideal biomarkers of ESCC. In addition, readers should note that some of the articles we referenced were published before the MISEV2018 guidelines were issued, which means the research methods they applied might not meet the standard of the guidelines. For example, few works failed to further validate sEV-specific markers, which is not recognized by the standards of EV isolation protocols in MISEV2018. Therefore, we hope readers accommodate the term “small extracellular vesicle” to replace “exosome”,

**Table 2:** Exosomal cargos as biomarkers for ESCC.

Type	Molecules	Origin	Potential Functions	Ref.
miRNA	miRNA -19b-3p	Serum	Distinguish ESCC patients from healthy individuals	(56)
	miRNA -21	Serum	Predict TNM stage	(117)
	miRNA -652-5p	Serum	Predict TNM stage, lymph node metastasis, and survival rate	(58)
	miRNA -339-5p	Serum	Predict radiotherapy sensitivity and survival rate	(64)
	miRNA -182	Serum	Distinguish ESCC patients from healthy individuals, predict TNM stage and survival rate	(118)
	miRNA -766-3p	Serum	Predict TNM stage and survival rate	(119)
	miRNA -103a-2-5p	Serum	Predict survival rate	(57)
	miRNA -93-5p	Serum	Predict survival rate	(126)
	chr 8-23234-3p,	Serum	Predict lymph node metastasis	(127)
	chr 1-17695-5p,			
	chr 8-2743-5p,			
	miRNA-432-5p			
lncRNA	POU3F3	Serum	Predict chemotherapy sensitivity and survival rate	(70)
	RP5-1092A11.2	Serum	Distinguish ESCC patients from esophagitis patients and from healthy individuals	(128)
	NR_039819	Serum	Distinguish ESCC patients from healthy individuals	(129)
	NR_036133			
	NR_003353			
	ENST00000442416.1			
	ENST00000416100.1			
	UCA1	Serum	Early diagnosis	(68)
	FMR1-AS1	Serum	Predict survival rate, especially in female ESCC patients	(65)
	POU3F3	Serum	Predict survival rate	(130)
circRNA	hsa-circ-0048117	Serum	Predict TNM stage	(71)
	hsa_circ_0026611	Serum	Predicted lymph node metastasis and survival rate	(120)
	hsa_circ_0001946	Serum	Predict recurrence and survival rate	(131)
	hsa_circ_0001946			
Other biomarkers	G-NchiRNA	Salivary	Reflect tumor burden, evaluate therapeutic response and predict survival rate	(132)
	uc.189	Serum	Evaluate lymph node metastasis	(133)
	Stathmin-1	Serum	Differentiate patients with ESCC from healthy individuals, and be associated with lymph node metastasis and advanced cancer stage	(125)

### Potential Application of Exosomes in Treatment of ESCC

In addition to having a diagnostic role in cancer, exosomes have potential use in disease therapy. The characteristic property in transferring selected payloads to recipient cells has translated into potential applications for treating many diseases, including cancer and cardiovascular diseases (134–136). However, recent articles published on the potential application of exosomes in the treatment of ESCC are sparse. Thus, this section mainly summarizes exosome-based therapy in other cancers and introduces the exosomes that may be potential targets for ESCC therapy in the future. Researchers harness engineered exosomes to deliver chemotherapeutic agents, achieving better performance than traditional vectors like liposomes (49). Theoretically, exosomes have the following advantages: First, the membrane structure of an exosome can protect pharmacological agents from degradation. Second, exosomes naturally exist in all biological fluids, and thus they can be well tolerated when introduced into the body. They can efficiently penetrate biological barriers and deliver targeted cargo with minimal immune clearance. Third, some exosomes have receptor-targeting features resulting from the heterogeneity of exosomal surface proteins, enabling targeted delivery of therapeutic agents for cancer. Last, because they are shed by all cells as part of their normal physiology, exosomes may induce less toxicity and minimize other adverse reactions even with repeated injection (137–140). Therefore, exosomes may have a bright future as nanocarriers for cancer treatment.

Currently, researchers are committed to designing exosomes to encapsulate therapeutic agents and conducting studies that yield valuable information about the application of exosomes for the administration of diseases. For example, gene-engineered exosome-thermosensitive liposomes can block the CD47 immune checkpoint and improve the macrophage-mediated elimination of cancer cells (30). Pan et al. indicated that urinary exosome-based engineered nanovectors could help deliver targeted homologous treatment in prostate cancer and may exemplify a novel, efficient, and facile therapy strategy (29). So far, evidence about the function of engineered exosomes in cancer therapy is primarily from cancers like gastric cancer or prostate cancer, not from ESCC; relevant data in ESCC are, at best, sparse. However, despite the many unanswered questions about their clinical application, exosomes show great potential to facilitate ESCC therapy. In addition, accumulating research has suggested that engineered exosomes *in vitro* can play a paramount role in different experimental settings, but more exploration is needed before these findings translate into clinical practice (141). Also, research must guarantee the homogeneity of exosomes by standardizing protocols for exosome isolation, preparation, and route of administration (8). Currently, inefficient isolation methods of exosomes cannot provide sufficient exosomes to meet cancer therapy requirements (142). Identifying ways to prevent exosomes from being taken up by other cells and to drive the engineered vehicles to targeted cells or organs remains a considerable challenge (143–145). Overall, engineered exosomes provide a promising therapeutic option for cancer treatment, although the utility of this strategy in clinical practice requires additional exploration.

## Conclusions and Perspectives

As referred to above, substantial evidence has delineated that exosomes and their inclusion, such as DNA, RNA, proteins, lipids, and other biological complexes, significantly affect cellular pathways and mediate pathophysiology behaviors, involving cell growth oncogenesis and tumor differentiation. EVs may act as biomarkers for early ESCC diagnosis, therapeutic monitoring, or prognosis evaluation. The association with exosomal cargos and disease state could be used in diagnostic and prognostic biomarkers for early ESCC, such as miRNA-21 was recognized as an exosome-derived small RNA superior to traditional tumor markers for early diagnosis of ESCC (117). It should be noted that a panel of miRNAs has delineated a higher sensitivity and specificity compared with a single miRNA, but researchers have to make a considerable effort to screen for miRNA panels that can be used in early diagnosis for ESCC (129, 130). In addition, there are some limitations in the clinical application of circulating exosome-related analysis. The separation and purification technology is mainly used in scientific research but rarely applied in clinical practice. Exosome-testing kits are developing, and some laboratory investigations and clinical studies on ESCC exosomes are ongoing or are forthcoming. For example, a newly developed commercial kit (ExoLutE^®^) utilizing the principle of size-exclusion spun column improves the efficiency and purity of circulating exosome separation compared to conventional kits (146).

Exosomes have been widely regarded as a promising carrier of anticancer drugs, which were proved in animal studies. If engineering exosomes that carried anticancer agents could be realized, the delivery of therapeutic agents by exosomes would make exosomes ideal vectors for cancer therapy (147). To date, evidence on the function of engineered exosomes in cancer therapy has mainly come from other cancers, such as pancreatic or prostate cancer (29, 148), and data on ESCC have been sparse at best. With the development of science and technology, large-scale clinical studies or trials on ESCC exosomes will undoubtedly be carried out. It is believed that more and more achievements on exosomes will be made and applied in ESCC clinical diagnosis and therapy soon.

## Author Contributions

Literature review and writing—original draft preparation: ZZ and SY. Writing—review and editing: AZ, XL, RF, and SZ. Supervision and funding acquisition: PL and GZ. All authors have read and agreed to the published version of the manuscript.

## Funding

This research was funded by the National Natural Science Foundation of China (no. 82070575), Beijing Natural Science Foundation (J180010), and Beijing Municipal Science & Technology Commission (Z191100006619080).

## Conflict of Interest

The authors declare that the research was conducted in the absence of any commercial or financial relationships that could be construed as a potential conflict of interest.

## Publisher’s Note

All claims expressed in this article are solely those of the authors and do not necessarily represent those of their affiliated organizations, or those of the publisher, the editors and the reviewers. Any product that may be evaluated in this article, or claim that may be made by its manufacturer, is not guaranteed or endorsed by the publisher.
